# Why Do Parotid Pleomorphic Adenomas Recur? A Systematic Review of Pathological and Surgical Variables

**DOI:** 10.3389/fsurg.2017.00026

**Published:** 2017-05-15

**Authors:** Pavel Dulguerov, Jelena Todic, Marc Pusztaszeri, Naif H. Alotaibi

**Affiliations:** ^1^Department of Otorhinolaryngology – Head and Neck Surgery, Geneva University Hospital, Geneva, Switzerland; ^2^Department of Pathology, Geneva University Hospital, Geneva, Switzerland

**Keywords:** pleomorphic adenoma, recurrence, parotidectomy, pathology, parotid tumors

## Abstract

**Background:**

The recurrence of pleomorphic adenoma (PA) has been extensively debated, mostly in relation to the extent of parotidectomy.

**Methods:**

A systematic review was undertaken to clarify the surgical and pathological variables related to PA recurrence. Inclusion criteria were as follows: English literature, and prospective or retrospective studies. Exclusion criteria were as follows: single case reports, reviews, and lack of PA recurrence data.

**Results:**

Pathology-related variables associated with recurrence include the histological subtype, the thickness and incompleteness of the tumor capsule, pseudopodia, and satellite nodules. Surgery-related variables associated with recurrence are the presence of intact margins and tumor puncture or spillage. Other factors are the size of the tumor and the age of patient. Myxoid subtypes of PA tend to have incomplete and thinner capsules and to recur more frequently. Surgical variables related to recurrence include positive margins and tumor spillage.

**Conclusion:**

Myxoid and/or large PA, especially in young patients, should be approached more cautiously to avoid recurrences.

## Introduction

Pleomorphic adenoma (PA) is the most common parotid gland neoplasm accounting for 50–60% of all parotid tumors (1, 2). The standard surgical treatment is an open parotidectomy I-V (3), which is associated with recurrence rates below 2–3% (4). By contrast, in earlier studies, recurrence rates following enucleation were as high as 40% (5, 6).

Despite this progress, the exact causes of PA recurrence have remained elusive. The widely held hypothesis is subtotal tumor removal due to inadequate surgery, while the characteristics of the tumor capsule or other histological aspects are rarely examined. We hypothesize here that the various reasons advanced for the recurrence of PA can be grouped into pathology-related (capsule thickness or lack of capsule, pseudopodia, satellite nodules, multi-centricity) and surgery-related (rupture of the tumor, spillage of tumor contents, insufficient margins of resection because of nerve branches, inadequate excision related to the type of surgery) factors. These clinicopathological and surgical features of PA are the basis for this review.

## Methods

The PubMed database was searched in December 2016 with the terms “pleomorphic adenoma” AND “recurrence” AND “parotid gland” AND “pathology” (search pathology) as well as with the terms “pleomorphic adenoma” AND “recurrence” AND “parotid gland” AND “surgery” (search surgery). Neither language nor time of publication restriction was placed.

The search “pathology” revealed 266 results, while the search surgery yielded 345. Of these, 210 publications were present in both searches, leaving 401 articles. The abstracts of these two searches were examined independently by two authors (Naif H. Alotaibi and Pavel Dulguerov) and any disagreement resolved through discussion.

We included randomized and non-randomized trials, and prospective and retrospective case series. Criteria for exclusion and the number of publications excluded are detailed in Table 1. After abstract-based exclusions, the remaining 79 articles were thoroughly evaluated. This led to the further exclusion of 49 additional publications for various reasons detailed in Table 1. References from the relevant articles were also searched, which yielded an additional 22 publications (1–3, 5–23).

**Table 1:** **Exclusion criteria and number of publications excluded**.

	Exclusion on abstracts	Exclusion after article review
Starting total	401	79
Cancer	70	
Case reports	24	2 (24, 25)
Evaluation	11	9 (26–34)
Metastatic pleomorphic adenoma (PA)	19	
Not PA (other benign histology)	13	
PA other locations (not parotid gland)	21	
Parotid tumors in general	50	3 (35–37)
Parotidectomy complications	4	5 (38–42)
Parotidectomy extent for PA	26	4 (43–46)
Parotidectomy techniques and outcome	27	1 (47)
Treatment of recurrent PA	38	3 (48–50)
No relevant data/reviews	19	22 (51–72)
Remaining publications	79	30

The outcome criteria were not predefined but were built as the literature review progressed. Any criterion that was found significant in one study was further searched in all relevant publications. Data were extracted and tabulated.

The heterogeneity in criteria evaluation and reporting, as well as the paucity of data precluded a meta-analysis and any statistical evaluation.

## Results

### Pathology-Related Variables

#### Pathological Subtype

**Figure 1:**
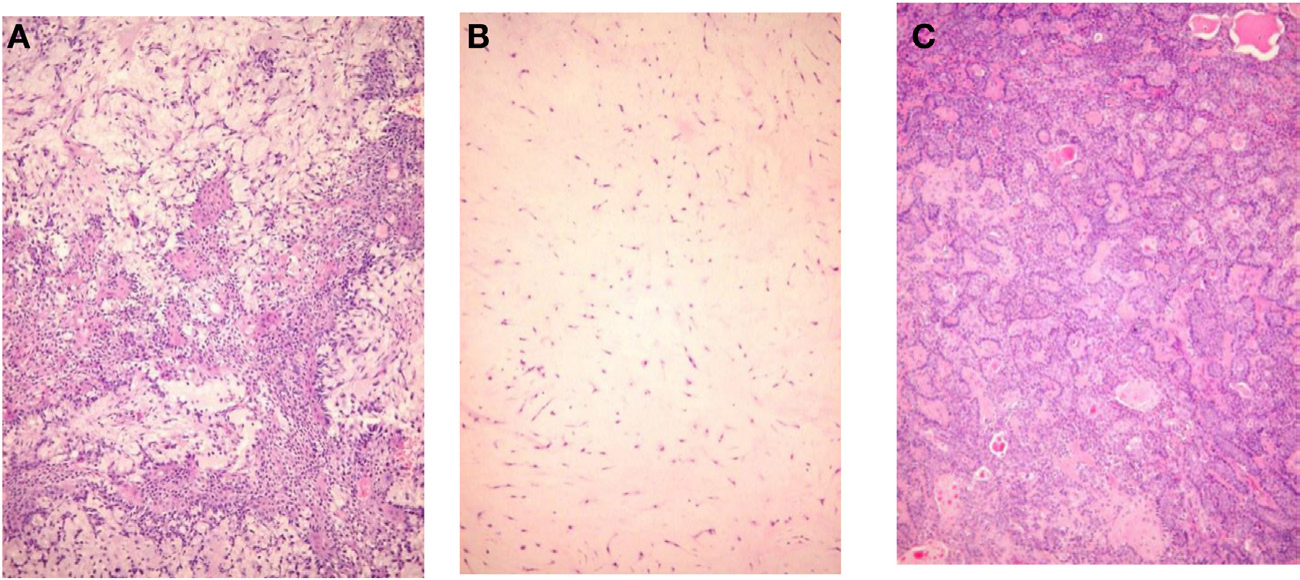
**Histological subtypes of pleomorphic adenoma**. **(A)** Classic type, **(B)** myxoid type, and **(C)** hypercellular type.

Pleomorphic adenoma draws its name from its architectural rather than cellular pleomorphism: epithelial and glandular elements intermingle with various amounts of mucoid, myxoid, or chondroid extracellular stroma. Seifert et al. (17) proposed a classification of PA based on relative composition of cellular and stroma components: hypocellular or stroma-rich or myxoid (50–80% of stroma), classical with balanced content (30–50% stroma), and hypercellular (<30% stroma). Myxoid PA account for more than half of cases, the classical subtype for a third, and the remaining 15% were hypercellular (Figure 1). The frequency distribution of these three subtypes varies in publications (Table 2), probably because their definition is not appreciated uniformly.

**Table 2:** **Distribution of pleomorphic adenoma subtypes**.

	Myxoid (%)	Mixed—classical (%)	Hypercellular (%)
Seifert et al. (17)	50	33	17
Naeim et al. (73)	39	27	33
Stennert et al. (18)	51	14	35
Webb and Eveson (19)	35	37	28
Zbären and Stauffer (74)	26	54	20
Park et al. (75)	19	75	6

Seifert et al. (17) noticed that recurrences were more frequent in the hypocellular subtype, an idea previously advanced by others (73, 76). By examining 31 recurrent PA, Stennert et al. (77) found a predominance of myxoid histology (25/31). This tendency was confirmed (58/65) in an update report from the same group (78), as well as by others (21, 75, 77–79).

#### Capsular Integrity

**Figure 2:**
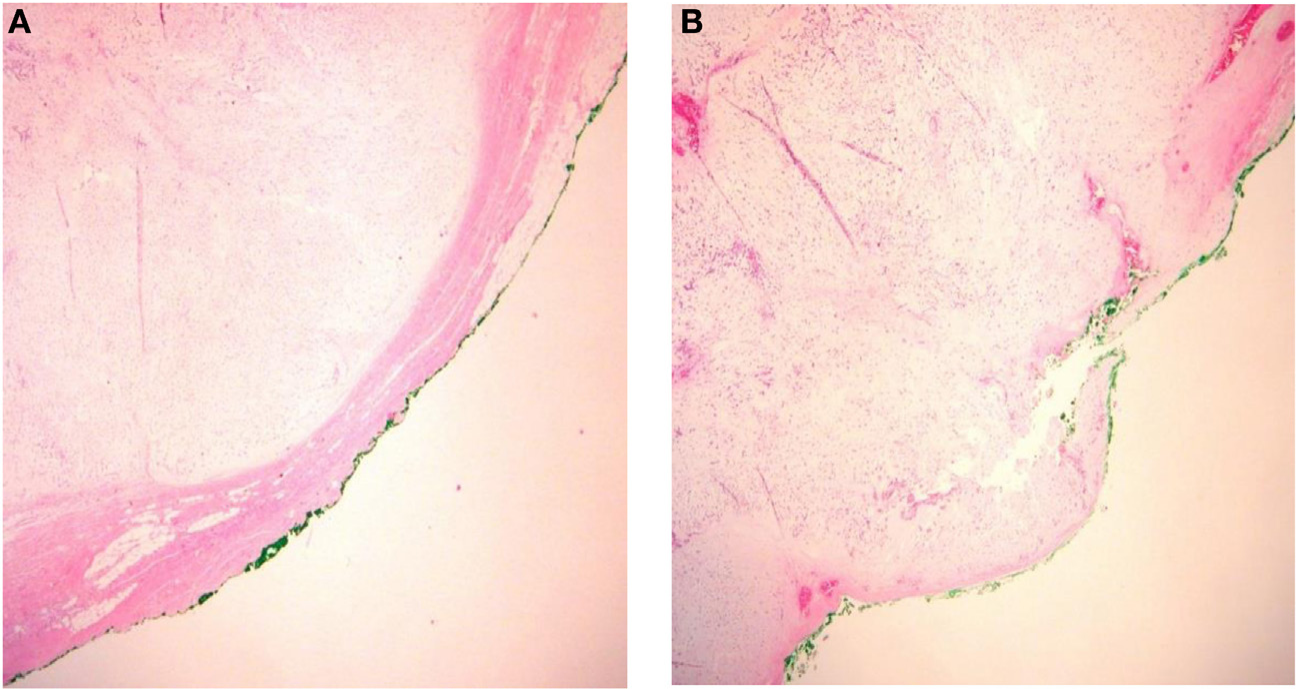
**Integrity of the pleomorphic adenoma capsule**. **(A)** Capsular integrity preserved and **(B)** ruptured capsula.

There is a consensus that, in the major salivary glands, PAs are enclosed by a layer of fibrous tissue termed a “capsule” (7). The presence of incomplete or bare capsule (Figure 2) as a source of recurrence was already discussed in the 1960s (10, 16).

Absence of encapsulation of PA is found in 20–50% of pathology specimen. A somewhat related parameter is the amount of tumor surface with an incomplete capsule, and this was estimated by Stennert et al. (18) to be about 4%.

Naeim et al. (73) were probably the first to relate incompleteness of encapsulation to the pathological subtype of PA: 69% of myxoid PA had incomplete capsules vs. 30% of classic subtypes, and 18% of hypercellular subtypes. Most authors (18, 19, 74, 75, 79, 80) confirm a larger incidence of incomplete capsules in myxoid PA, although the statistical differences are not always significant. Stennert et al. (18) describe more extensive areas of incomplete capsule in the myxoid type, up to 28% of the whole tumor circumference.

The relationship between incomplete encapsulation and recurrence is summarized in Table 3. Although the data are sparse and often not statistically tested, in general, there is a trend for higher incidence of incomplete capsules in recurrent PA.

**Table 3:** **Pathological and surgical variables related to recurrence of pleomorphic adenoma (PA)**.

Reference	Lack of capsule^a^ (%)	Pseudopodia^a^ (%)	Satellite nodules^a^ (%)	Multi-centricity^a^ (%)	Exposed capsule^a^ (%)	Gross specimen damage (%)	Tumor puncture^a^ (%)	Tumor spillage^a^ (%)	Margins^a^
Danovan and Conley (8)	21				40	27			
Goudot et al. (79)	4/14								
Lam et al. (14)	33			0	100	27			
Natvig and Soberg (81)					36			2/33	
Henriksson et al. (13)		8/55					4/7	12/22	
Stennert et al. (18, 77)	46/66	28	28						
Webb and Eveson (19)		12		0	81	5.5			
Ghosh et al. (11)								5/11	1.8%/18%^b^
Paris et al. (82)		53/60		0					
Zbären and Stauffer (74)	33	40	13	0	80				
Orita et al. (83)			3	1					
Riad et al. (84)				0			11/100	1/57	1.3/6 mm^c^
Park et al. (75)	58/70	53/60	10/60				4/30	4/30	11%/50%

*^a^Incidence of characteristic in non-recurrent PA/recurrent PA*.

*^b^>1/<1 mm*.

*^c^Average margin in non-recurrent PA/recurrent PA*.

#### Capsular Thickness

Thickness of the capsule of PA was found to vary from 5 to 250 µm by Stennert et al. (18). Although the details are not provided, myxoid PA is described as being at the lower end (5 μm) of the spectrum and cellular PA at the higher one (250 μm). Similar results were found by Naeim et al. (73), Webb and Eveson (19), and Zbären and Stauffer (74). There is no study examining capsular thickness and recurrence.

#### Pseudopodia

**Figure 3:**
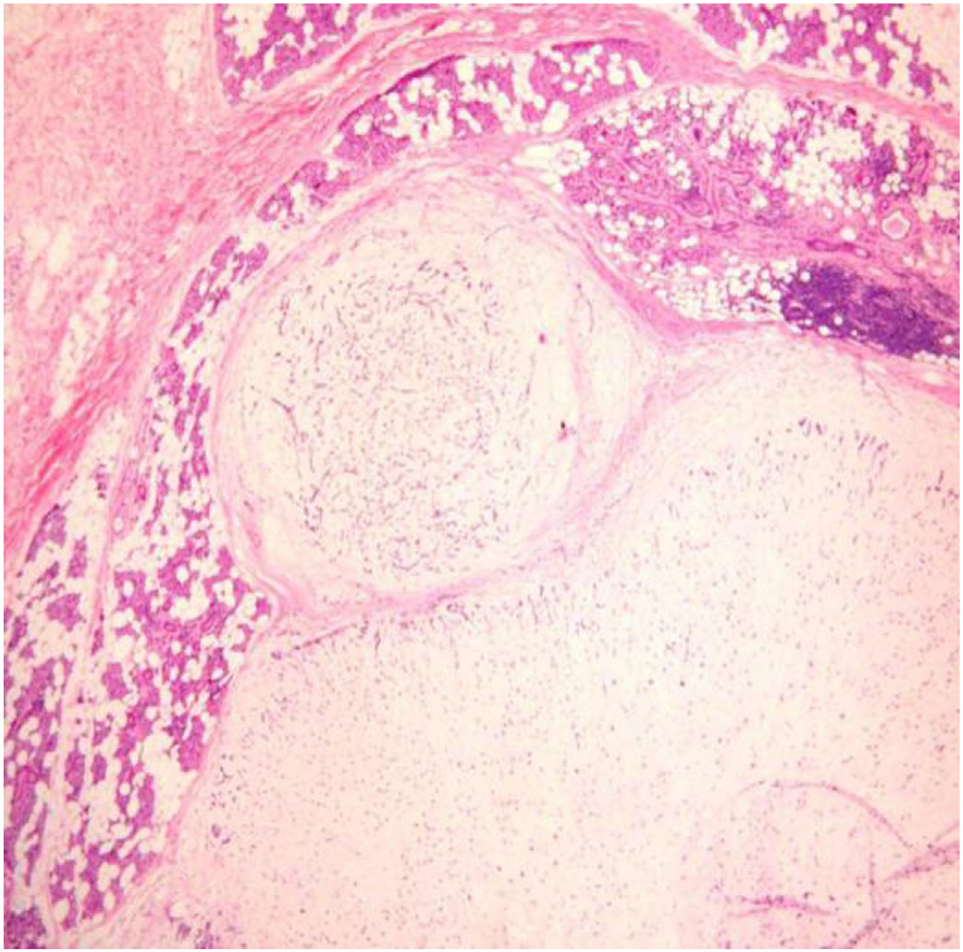
**Pseudopodia**.

Pseudopodia are tumor nodules bulging from at the tumor edges and separated by fibrous tissue from the main tumor mass but still localized within the main tumor capsule (Figure 3). Pseudopodia were recognized and advanced as a cause of PA recurrence already in the 1950s by Patey and Thackray (16).

Pseudopodia were found in 28% of PA in Stennert’s series (18), in 40% of PA by Zbären and Stauffer (74), and in 54% by Park et al. (75). No clear correlation was found between pseudopodia and PA subtype (18, 74, 75).

Henriksson et al. (13) observed pseudopodia in 5 of 9 (55%) PA with subsequent recurrence and in 16 of 197 (8%) without subsequent recurrence, although this series was not limited to the parotid gland. No such difference was found by Park et al. (75).

#### Satellite Nodules

**Figure 4:**
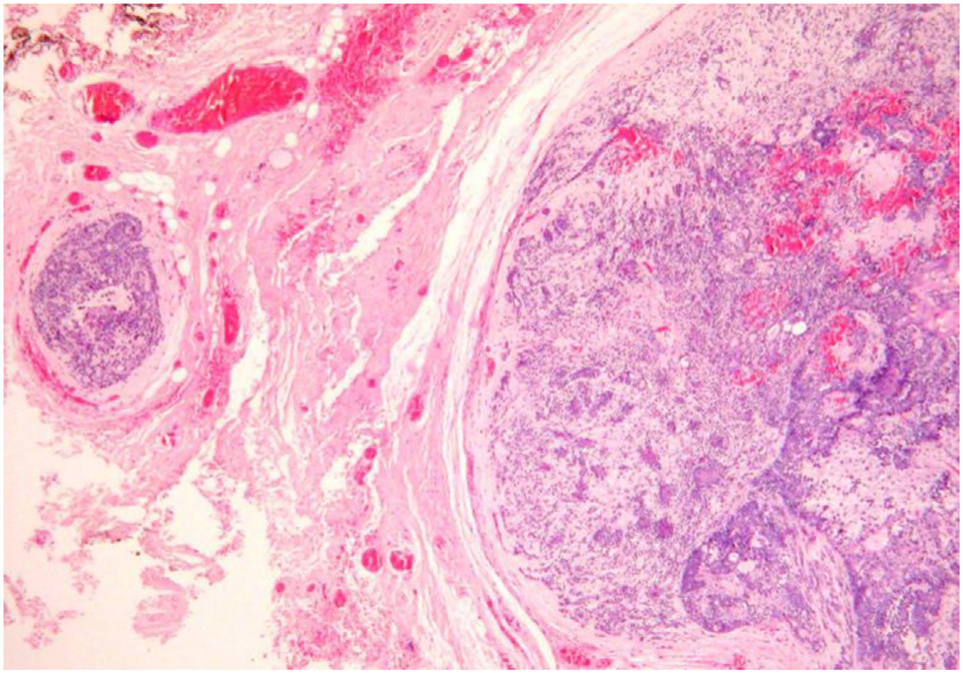
**Satellite nodules**.

Satellite nodules as distinct tumor nodules in the vicinity of the main tumor lump but separated from it by salivary or fat tissue without any direct connection being traceable on serial histopathology sections (Figure 4). Orita et al. (83) and Li et al. (80) evaluated the distance between the main tumor and satellite nodules and found a maximum of 5.0 and 8.5 mm, respectively.

When satellite nodules extend to such a distance, one might wonder if PA could be multifocal (9). Most serial section studies have failed to confirm this hypothesis (14, 16, 19, 74, 84), although Orita et al. (83) found one case (1%) of multifocal PA.

These satellite nodules were found in 15% (74, 75) to 28% (18) of PA. There is no correlation between histological PA subtype and occurrence of satellite nodules (74, 75, 80).

Park et al. (75) found an increased prevalence of satellite nodules in patients with recurrent PA: 6 of the 10 patients (60%) vs. only 10% in non-recurrent PA.

#### Tumor Size

Tumor size could be included as a pathological, surgery-related, or patient variable. More recently, this relation between PA tumor size and capsular integrity was examined by Li et al. (80): larger tumors (>4 cm) more often exhibited incomplete capsules, although the difference was not significant. Similar findings are described by Webb and Eveson (19) and Zbären and Stauffer (74).

A significant correlation between the size of tumor and satellite nodules was found, with larger PA exhibiting more often satellite nodules (74, 75, 80).

In Riad et al. (84) series, the mean PA diameter was 32.8 mm and recurrence of PA was related to tumor size, with a mean tumor diameter of 30 mm in non-recurrent PA and of 43 mm in the recurrent PA. Similarly, in Ghosh et al. (11) series, all recurrences occurred in tumors larger than 2.5 cm. On the other side, Henriksson et al. (13) could not correlate tumor size and recurrence.

#### Other Pathological Variables

Bankamp and Bierhoff (7) evaluated the proliferation index of recurrent and non-recurrent PA by the MIB-1 antibody immunohistochemistry against the cell proliferation-associated nuclear antigen (Ki-67 antigen): a statistically significant difference was present with a proliferation index of 4.67 ± 3.14 for recurrent PA and 2.34 ± 1.83 non-recurrent PA. Higher MIB-1 expression was also found by Glas et al. (85) and Stennert et al. (77) but the differences were not significant.

The presence of multiples nodules in recurrent PA prompted Stennert et al. (77) to suspect a lymphatic spread. This hypothesis was tested by immunohistochemistry for vascular endothelial factors C and D (VEGF-C and VEGF-D), which are ligands for endothelial tyrosine kinase receptor VEGFR-3, known to primarily mediate lymphangiogenesis. The results are inconclusive (20) and do not support the hypothesis of local lymphangiogenic spread.

A higher expression of mucin 1 (MUC1) glycoprotein and especially of the MUC1/DF3 glycoform by immunostaining was found in recurrent PA and was the only variable significant in multivariate analysis (21). Only 8% of non-recurrent PA showed some immunostaining for MUC1/DF3 (21). Other studies have confirmed the progressively increased staining for MUC1/DF3 from normal salivary gland to non-recurrent PA, recurrent PA, and carcinoma ex PA (22, 23). In addition, the staining pattern appears different in recurrent and non-recurrent PA (22).

### Surgery-Related Variables

#### Resection Margin

Riad et al. (84) collected prospective data on 180 parotidectomies: 162 new tumors and 18 recurrent PA. The resected margins ranged between 0 and 26 mm, with a mean of 5.8 mm. Tumor recurrence was inversely related to the safety margin with an average of 1.3 mm in recurrent PA and of 6.0 mm in non-recurrent PA. Furthermore, the safety margin was inversely correlated with tumor size.

Ghosh et al. (11) reviewed the slides of 83 PA with prolonged follow-up (mean 12.5 years) and noted 6% recurrences. They classified the specimen margins (0; <1 mm; >1 mm) and amount of close margins relative to the specimen circumference (focal as <10% and wide as >10%). All recurrences were in the widely exposed capsule group, and most recurrences were found in margins either 0 or <1 mm (18%) vs. 1.8% if larger margins were obtained.

Positive margins were also seen as source of recurrence by Buchman et al. (86).

#### Tumor Puncture and Spillage

Tumor puncture is described as a breach in the tumor capsule during surgery and described in the pathological report. Spillage consists of spread in the surgical field of tumor content.

The incidence of tumor puncture during parotidectomy is unclear, although Henriksson et al. (13) describe an incidence of 14%. In the study of Riad et al. (84), all the 7 tumors that recurred were punctured (100%), while there was an incidence of 11% of puncture in non-recurrent PA. Spillage was reported in seven patients and four developed a recurrence (80%). Park et al. (75) conclude that the risk of recurrence is more than 14-fold higher (4 vs. 30%) if the tumor had ruptured during the surgery.

High incidence of recurrences with tumor puncture was also reported by Henriksson et al. (13) (7 vs. 4%), although the difference was not significant. High incidence of recurrences with tumor spillage were also reported by Ghosh et al. (11) (11 vs. 5%), although the difference was not significant. Buchman et al. (86) found that recurrence did not necessarily follow tumor spillage, although different treatment options including radiation were employed preventively to avoid recurrence.

### Patient-Related Variables

The younger age at initial presentation of patients who later present a recurrence is well established (15, 21, 48, 78, 87, 88): patients who will later exhibit a recurrent PA tend to be 20 years younger at initial presentation. However, this relationship was not confirmed in some series (84).

## Discussion

Recurrence of PA is a complex problem, occurring 2–15 years after the initial parotidectomy (78, 87) and in relatively young patents (15, 48, 78, 87). The recurrence is often multinodular (50–100%) (77, 87) and associated with an increased rate of post-operative complications, especially facial nerve paralysis (2–20%) (78, 87), further recurrences (77, 78), and malignant degeneration (0–16%) (87–90). Even expert surgeons consider parotidectomy of recurrent PA as one of the most challenging head and neck procedures.

The relative low incidence of recurrent PA and the necessity of a prolong follow-up, associated with the lack of national or international databases, has hampered progress. A recent publication from a Dutch registry covering close to 5,500 patients followed for 15 years and treated with formal parotidectomy (3) puts the incidence of recurrent PA to 2.9% and the rate of malignant transformation to 3.3% (88).

Beyond incidence, the time frame between initial surgery and recurrence implies that the treatment of the recurrence is often performed by a different surgeon and often in a different institution. As a result, the pathology slides from the initial parotidectomy are often unavailable for analysis. Furthermore, surgeons tend to blame recurrence on previously inadequate procedure and little progress has been achieved on the exact causes of PA recurrence. Conceptually, tumor reappearance to inadequate initial resection could be viewed as PA persistence rather than PA recurrence.

Despite the lack of randomized studies and the paucity of prospective studies, the available data in this systematic review seem to point toward a series of coherent arguments. Myxoid subtypes of PA tend to have incomplete and thinner capsules and to recur more frequently. Larger PA tend not only to exhibit incomplete capsules but in addition are associated with more numerous satellite nodules. Larger tumors seem, in most studies, to be associated with more frequent recurrences. When surgical variables are examined, it seems that positive margins and tumor spillage are linked to recurrences. In possibly the only study with some statistical power, Park et al. (75) demonstrated significant differences for satellite nodules, positive margins, and tumor spillage in univariate analysis and for positive margins, and tumor spillage in multivariate analysis.

Because of the measured spread of satellite nodules, Li et al. (80) recommend margins of healthy parotid tissue of about 1 cm. Unfortunately, this requirement seems impossible to fulfill because tumors often abut the facial nerve or its branches as already demonstrated by Danovan and Conley (8) and others (see Table 3). If medial capsular exposure is sometimes unavoidable, there is no good reason to obtain an exposed capsule on the lateral, inferior, and even superior aspects of a PA. Sternocleidomastoid, SMAS, and even skin can be sacrificed to obtain adequate margins, and this can be performed without compromising both functional and esthetic aspects of parotidectomy.

We purposefully tried to avoid the ongoing controversy of the extent of parotidectomy in PA (4, 91–96). As expected, capsular exposure is much more frequent in extracapsular dissection (4, 91, 95). While low rates of recurrence with extracapsular dissection have been published by experienced groups in highly selected cases, series with elevated rate of recurrence also exist (90, 95–97) and a systematic review concluded to a higher recurrence rate of PA with this procedure (92). Probably limited resections, if recommendable at all, should be limited to selected cases with small tumor size and not of the myxoid subtype. If sufficient margins cannot be achieved, the procedure should be converted to a formal parotidectomy.

## Conclusion

Recurrence of PAs seem to be increased in the myxoid PA subtype in which the capsule is more often thinner and incomplete. Recurrences seem also more frequent in larger tumors, which not only exhibit incomplete capsules but are also associated with more numerous satellite nodules. Surgical variables related to recurrence include positive margins and tumor spillage.

## Author Contributions

All the authors: design of the work; data acquisition, analysis, interpretation, draft contribution, and approval of the final version to be published; agreement to be accountable for all aspects of the work in ensuring that questions related to the accuracy or integrity of any part of the work are appropriately investigated and resolved.

## Conflict of Interest Statement

The authors declare that the research was conducted in the absence of any commercial or financial relationships that could be construed as a potential conflict of interest.
